# Optimizing genetic ancestry adjustment in DNA methylation studies: a comparative analysis of approaches

**DOI:** 10.1186/s13072-025-00627-0

**Published:** 2025-10-14

**Authors:** Kira D. Höffler, Seyma Katrinli, Matthew W. Halvorsen, Anne-Kristin Stavrum, Kevin S. O’Connell, Alexey Shadrin, Srdjan Djurovic, Ole A. Andreassen, James J. Crowley, Jan Haavik, Kristen Hagen, Gerd Kvale, Kerry Ressler, Bjarne Hansen, Jair C. Soares, Gabriel R. Fries, Alicia K. Smith, Stéphanie Le Hellard

**Affiliations:** 1 Department for Clinical Science, University of Bergen, Bergen, Norway; 2 Bergen Center for Brain Plasticity, Division of Psychiatry, Haukeland University Hospital, Bergen, Norway; 3 Dr. Einar Martens Research Group for Biological Psychiatry, Department of Medical Genetics, Haukeland University Hospital, Bergen, Norway; 4 Department of Psychiatry, McLean Hospital, Harvard Medical School, Belmont, MA USA; 5 Department of Gynecology and Obstetrics, Emory University, Atlanta, GA USA; 6 Department of Genetics, University of North Carolina at Chapel Hill, Chapel Hill, NC USA; 7 Center for Precision Psychiatry, Division of Mental Health and Addiction, Oslo University Hospital and Institute of Clinical Medicine, University of Oslo, Oslo, Norway; 8 Department of Medical Genetics, Oslo University Hospital and University of Oslo, Oslo, Norway; 9 Section for Precision Psychiatry, Oslo University Hospital, Oslo, Norway; 10 Department of Psychiatry, University of North Carolina at Chapel Hill, Chapel Hill, NC USA; 11 Department of Biomedicine, University of Bergen, Bergen, Norway; 12 Department of Psychiatry, Møre and Romsdal Hospital Trust, Molde, Norway; 13 Department of Mental Health, Norwegian University of Science and Technology, Trondheim, Norway; 14 Department of Clinical Psychology, University of Bergen, Bergen, Norway; 15 Center for Crisis Psychology, University of Bergen, Bergen, Norway; 16 Faillace Department of Psychiatry and Behavioral Sciences, McGovern Medical School, The University of Texas Health Science Center at Houston, Houston, TX USA; 17 Department of Psychiatry and Behavioral Sciences, Emory University, Atlanta, GA USA

**Keywords:** Epigenetics, DNA methylation, Ancestry, EWAS, MWAS

## Abstract

**Background:**

Genetic ancestry is an important factor to account for in DNA methylation studies because genetic variation influences DNA methylation patterns. One approach uses principal components (PCs) calculated from CpG sites that overlap with common SNPs to adjust for ancestry when genotyping data is not available. However, this method does not remove technical and biological variations, such as sex and age, prior to calculating the PCs. The first PC is therefore often associated with factors other than ancestry.

**Methods:**

We developed and adapted the adapted *EpiAnceR+* approach, which includes (1) residualizing the CpG data overlapping with common SNPs for control probe PCs, sex, age, and cell type proportions to remove the effects of technical and biological factors, and (2) integrating the residualized data with genotype calls from the SNP probes (commonly referred to as rs probes) present on the arrays, before calculating PCs and evaluated the clustering ability and relationship to genetic ancestry.

**Results:**

The PCs generated by *EpiAnceR+* led to improved clustering for repeated samples from the same individual and stronger associations with genetic ancestry groups predicted from genotype information compared to the original approach. *EpiAnceR+* also outperformed the use of DNA methylation PCs or surrogate variables for ancestry adjustment.

**Conclusions:**

We show that the *EpiAnceR*+ approach improves the adjustment for genetic ancestry in DNA methylation studies. *EpiAnceR+* can be integrated into existing R pipelines for commercial methylation arrays, such as 450 K, EPIC v1, and EPIC v2. The code is available on GitHub (https://github.com/KiraHoeffler/EpiAnceR).

**Supplementary Information:**

The online version contains supplementary material available at 10.1186/s13072-025-00627-0.

## Background

Genetic ancestry is an important confounding factor in DNA methylation studies and should ideally be adjusted for using principal components (PCs) derived from genetic information (i.e. genotypes). However, when genotype data is not available for all study participants, self-reported ancestry data is commonly used to adjust for ancestry in DNA methylation studies, often leading to the exclusion of non-Europeans and individuals with mixed genetic backgrounds, and to sub-optimal correction. This practice is problematic due to the historical underrepresentation of non-Europeans in epigenetic research [1] and the inherent flaws of self-reported data, which fail to capture the continuous nature of genetic variation and inadequately address the complexity of mixed ancestry backgrounds. To ensure the generalizability of research findings, DNA methylation studies should avoid excluding individuals based on genetic background.

Two main methods are available to adjust for genetic ancestry in DNA methylation studies lacking genotyping data. The method by Barfield et al. from 2014 [2] calculates PCs from DNA methylation data of CpGs near or overlapping single nucleotide polymorphisms (SNPs), selected based on the 1000 Genomes Project [3] data. However, this approach does not account for technical and biological factors such as sex, age, and cell type proportions, resulting in the first ancestry PC not being regularly associated with genetic ancestry [2] and potentially introducing multicollinearity with other adjusted factors in the final model. The EPISTRUCTURE [4] approach calculates PCs from DNA methylation of CpGs highly correlated with cis-located SNP probes while considering cell-type composition. This method is not easily integrated into existing R-based pipelines because it is a Python program and requires several steps of input file adaptation. Additionally, it has not been updated since 2017 and does not accommodate the EPIC v2 array. Other methods based on DNA methylation, such as Surrogate Variables and methylation PCs from the whole array data, do not specifically adjust for ancestry.

Therefore, there is a need for a reliable method to adjust for ancestry in DNA methylation studies where genotype data is not available that can easily be incorporated into existing pipelines. Our aim was to provide such a method by improving the approach by Barfield et al.'s [2] through the use of methylation data from CpGs overlapping with SNP probes that are residualized for technical and biological factors (i.e. cell type proportion PCs, sex, age, and control probe PCs) and information from genotyping SNP rs probes on the array. We hypothesized that ancestry PCs from our adapted approach would result in better clustering of genetically predicted ancestry groups and repeated samples from the same individual, stronger associations with genetic ancestry, and stronger correlations with genetic PCs. We tested the performance of our approach against the existing one using two Norwegian cohorts (BCBP-OCD and TOP [5]– [7]) and two US-American cohorts: UTHealth Houston [8] and Grady Trauma Project (GTP) [9]. An easy-to-use function for our adapted approach is provided on GitHub, along with detailed guidance on parameter settings and input file structure for implementation (https://github.com/KiraHoeffler/EpiAnceR). 

## Methods

### Tested cohorts

The different approaches were tested in two cohorts from Norway and two cohorts from the United States, including individuals with available DNA methylation data and ancestry information (either genetically predicted or self-reported, Table 1). The inclusion criteria were passing DNA methylation quality control and, if available, genotyping quality control. Samples from individuals with mixed ancestry (based on the available data, as described in the genetic ancestry prediction section) were excluded from performance testing solely for methodological reasons, because evaluating classification accuracy requires clearly defined ancestry groups, and admixed individuals typically fall between clusters. This is not a limitation of *EpiAnceR+* , which produces continuous ancestry PCs that capture both discrete and admixed variation, making it fully applicable to individuals with mixed ancestry.

**Table 1:** Cohorts on which the approaches were tested

	BCBP-OCD (n = 522)	TOP (n = 313)	Grady trauma project (GTP) (n = 740)	UTHealth Houston (n = 226)
Collection area	8 different clinics across Norway	Oslo region in Norway	Grady Memorial Hospital, Atlanta, GA	Greater Houston area
Main phenotype	389 individuals with OCD, 133 healthy controls	189 individuals with schizophrenia spectrum disorder or bipolar disorder, 4 individuals in prodromal phase, 120 controls		154 individuals with bipolar disorder, 72 non-psychiatric controls
Number of samples	Up to 3 samples per individual	Up to 2 samples per individual	1 sample per individual	1 sample per individual
Ancestry grouping	Predicted from genotyping data	Predicted from genotyping data	Harmonized using self-report and genotype data prediction	Self-reported
Ancestry of individuals (total number of samples, including repeated samples)	EUR: 510 (1220) EAS: 5 (15) SAS: 5 (13) AFR: 1 (3) AMR: 1 (3)	EUR: 250* SAS: 39 (41) EAS: 17 (18) AFR: 7 ARA: 5 AMR: 1	EUR: 24 AFR: 716	EUR: 87 EAS/SAS: 14 AFR: 76 AMR: 2 LAT: 46 PI: 1
%female	69.73%	48.24%	72.03%	69.02%
Mean age in years (± SD)	31.41 (± 9.53)	31.06 (± 9.12)	42.27 (± 12.24)	35.5 (± 11.01)
Tissue	Saliva	Whole blood	Whole blood	Whole blood
Array	EPIC v2	EPIC v1	EPIC v1	EPIC v1

*AS* Asian, *EAS* East Asian, *AFR* African, *AMR* American, *ARA* Arabian, *BCBP* Bergen Center for Brain Plasticity, *EUR* European, *LA* Latin American, *OCD* obsessive–compulsive disorder, *PI* Pacific Islander, *SAS* South Asian

*250 European samples were randomly selected from the whole TOP cohort.

### DNA methylation

In the BCBP-OCD cohort, DNA methylation was measured using the Illumina EPIC v2 array at Life&Brain GmbH in Bonn, Germany. Samples were excluded if they showed: mismatches between reported and predicted sex, outliers in the *minfi* [10] sex plot, genotype mismatches between repeated samples [11], low bisulfite conversion efficiency (< 80%) calculated with the *wateRmelon* [12] package, more than 5% missing data (after applying a detection p value threshold of 10E^−16^), or outlier patterns in beta value distribution plots [10]. Saliva cell type proportions were estimated using HEpiDISH [13] with the RPC method. The centEpiFibFatIC.m dataset, excluding fat, was used as the main reference, while the centBloodSub.m dataset functioned as the secondary reference [13].

In the TOP cohort [5]– [7], DNA methylation was measured using the Illumina EPIC v1 array at life and brain GmbH in Bonn, Germany, and quality controlled in three batches. Samples were excluded if at least 1% of sites had a detection *p*-value > 0.01, if SNP genotypes from the rs probes on the EPIC array did not match genotype data from the same samples, or there was a discrepancy between the sex predicted from DNA methylation data and the reported sex [5]. The blood cell type proportions were calculated using the estimateCellCounts2 function from FlowSorted.Blood.EPIC [14] package.

In the GTP cohort [9], DNA methylation was measured using the Illumina EPIC v1 array and quality controlled using the Psychiatric Genomics Consortium (PGC)-PTSD Epigenetics pipeline [15] (available at https://github.com/PGC-PTSD-EWAS/EPIC_QC). Briefly, samples were excluded if their probe detection call rates were below 90% (after applying a detection p value threshold of 0.01) or if their average intensity values were either less than half of the overall sample mean or below 2000 arbitrary units (AU). Blood cell composition - including CD8 + T cells, CD4 + T cells, natural killer cells, B cells, monocytes, and neutrophils - was estimated using the robust partial correlation (RPC) method in *Epidish* [16], utilizing a reference dataset specific to the EPIC array [17].

In the UTHealth Houston cohort [8], DNA methylation was measured using the Illumina EPIC v1 array. Samples with a mean detection p-value below 0.05 were set to be excluded; however, no samples met this criterion, so none were excluded. White blood cell count proportions (CD8 + T cells, CD4 + T cells, natural killer cells, B cells, monocytes, and granulocytes) were estimated using the Houseman algorithm [18].

### Genetic ancestry prediction

*BCBP-OCD:* Genotyping data for the BCBP-OCD cohort were processed following the quality control procedures described in the latest PGC OCD GWAS study [19]. Ancestry prediction was performed as outlined by Halvorsen et al. [20], using principal component analysis and a random forest classifier trained on 1000 Genomes Project Phase 3 data [3].

*TOP:* Genotyping data and quality control procedures for the TOP cohort are described in detail in previous work [21]. Ancestry was inferred from genotyping data using a Random Forest classifier trained on population reference data from the 1000 Genomes Project [3], as previously described [5].

*GTP: * genotyping was performed using Illumina Omni-Quad 1 M, and genotypes were called in Illumina’s GenomeStudio. Genotype data quality control and calculation of PCsoty were performed according to the PGC guidelines [22]. Ancestry was predicted as described by Nievergelt et al. [22].

*UTHealth Houston:* Ancestry information was assessed by self-report, as genotyping data were not available.

### EpiAnceR+ approach

The *EpiAnceR+* approach uses the *minfi* [10], *ChAMP* [23], and *wateRmelon* [12] packages in R (developed in version 4.4.0) to generate “ancestry PCs”, which can be incorporated into final analysis models for samples that have passed general quality control. The approach consists of two main functions: ancestry_info() and ancestry_PCA().

#### ancestry_info() function

The ancestry_info() function uses an RGset that has been background-corrected using the bg.correct.illumina() [10] function. Data from control probes, SNP rs probes, bead counts, and detection p-values are extracted, as well as intensities of type I probes (split into green and red channels) and type II probes. A detection p-value threshold of 10E^−16^ is applied, setting values above this threshold as missing. The intensities are filtered to include only CpGs overlapping with SNPs, referred to as SNP0bp probes.

The probes are selected using the same underlying criteria across all arrays, though the implementation depends on the structure of the available array-specific annotation:

- 450 K array: from the *IlluminaHumanMethylation450kanno.ilmn12.hg19* [24] annotations, selecting SNPs probes from CpG_rs column overlapping with CpG sites filtered based on minor allele frequency (MAF) ≥ 0.05.
- EPIC v1 array: from the *Pidsley *et al*. * [25] and *Zhang *et al*. * [26] annotations, selecting probes in either or both publications overlapping with SNPs (distance = 0) with a MAF ≥ 0.05
- EPIC v2 array: from annotations provided by Illumina [27], selecting probes overlapping with SNPs (distance = 0) with a MAF ≥ 0.05

The number of selected ancestry-informative probes was 7295 for the 450 K array, 3622 for the EPIC v1 array, and 2,674 for the EPIC v2 array. Probes with low bead counts (where > 5% of samples have a bead count below 3) or low call rates (where > 10% of samples have missing data) are excluded. After filtering, the intensity values are quantile normalized, and beta values are calculated. Missing values are imputed using the champ.impute() [23] function. Beta values for the SNP0bp probes are then residualized to adjust for cell count PCs, sex, age, and ten PCs derived from control probes, which capture a broad range of technical variation, including slide, array position, scanner effects, DNA quality, and bisulfite conversion efficiency. This residualized data is referred to as *EpiAnceR*. Finally, the residualized SNP0bp data is combined with the genotype calls from rs SNP probes, which is referred to as *EpiAnceR+* .

#### ancestry_PCA() function

The ancestry_PCA() function takes the processed data from ancestry_info() and uses it to calculate ancestry PCs. These PCs are intended to be included in analysis models as covariates to control for ancestry-related variation.

For further details and access to the code, please refer to the code on GitHub (https://github.com/KiraHoeffler/EpiAnceR).

### Performance tests

Three versions of ancestry PCs were compared: the original Barfield [2] et al. approach (*EpiAncOrig*), the adapted approach after only performing the residualization step (*EpiAnceR*), and the further adapted approach combining the residualised data with called genotypes from rs probes (*EpiAnceR+*). To ensure comparability across approaches, the PCA results were min–max scaled to a range between 0 and 1.

Associations between ancestry PCs and ancestry group were tested using ANOVA when assumptions of normality and variance homogeneity were met. Otherwise, Kruskal–Wallis tests with the *rstatix* [2] package were used.

To evaluate clustering performance, silhouette scores [28], which range from -1 to 1 (with higher values indicating better clustering), were calculated using the *cluster* [29] package. These scores were used to evaluate clustering performance at both the ancestry group level and the individual (repeated samples) level.

In addition, three-dimensional (3D) centroids of both ancestry group and individual (repeated samples) clusters were calculated. Cluster density was assessed by calculating the mean Euclidean 3D distance from each sample to the centroid of its respective group. To determine the separation between ancestry group clusters, a distance matrix was generated to measure the distances between the centroids of different ancestry groups.

In the BCBP-OCD cohort, the absolute correlation between the first three ancestry PCs and the first three genotyping PCs was calculated to evaluate how effectively the methylation-based PCs capture the genetic ancestry background.

Visualization of results was performed using *ggplot2* [30], *cowplot* [31], *patchwork* [32], *plotly* [33], *htmlwidgets* [34], and *corrplot* [35] packages.

### Comparison with DNA methylation PCs and surrogate variables

In the BCBP-OCD sample, we compared the performance of *EpiAnceR+* with that of the first three DNA methylation PCs and the first three surrogate variables (SVs), using the same evaluation approach described above. DNA methylation PCs were calculated from normalized M-values from autosomal probes.

Surrogate variable analysis was performed on the M-values. The full model included case–control status, age, sex, two cell type PCs, and ten control probe PCs. The null model excluded case–control status.

### Ancestry group associations with technical and biological adjustment factors

Associations between ancestry groups and the factors used to residualize SNP0bp data were tested using either linear models for datasets with two ancestry groups or ANOVA when multiple ancestry groups were present (with a common p-value for the effect across all groups).

For control probe PCs and cell type PCs in datasets with repeated samples, linear mixed-effect models were employed to test the association with the ancestry groups. These models included a random intercept to account for repeated measures from the same individual. Two models were compared: (A) a full model that included ancestry group as a predictor and the PCs as outcome; (B) a null model that excluded ancestry group. A likelihood ratio test was performed to determine if the full model fit the data significantly better than the null model.

Additionally, to assess whether residualized biological and technical covariates confounded our *EpiAnceR+* PCs, we performed linear regression and partial R^2^ analyses in the GTP cohort. For each of the top three *EpiAnceR+* PCs, we quantified the unique variance explained by (i) the top three genotype-derived ancestry PCs, (ii) residualized covariates (sex, age, top 10 control probe PCs, and 6 cell proportion PCs), and (iii) both sets together. Unique variance was computed using partial R^2^, representing the proportion of variance in each *EpiAnceR+* PC explained by one set of predictors after accounting for the other.

### Practical example

In the UTHealth Houston cohort, we conducted an analysis of bipolar case–control status, adjusting for sex, age, smoking status, five cell type proportion PCs, five control PCs, and 0, 2, 5, or 10 ancestry PCs calculated with *EpiAnceR*+ . This setup allowed us to compare the impact of including different numbers of ancestry PCs on the genomic inflation factor (λ).

## Results

We evaluated the performance of ancestry PCs in capturing genetic ancestry, comparing the original approach by Barfield et al. [2] (*EpiAncOrig*) with two adapted versions. The first adaptation step involved residualizing CpG data overlapping with SNPs to adjust for technical and biological confounders (*EpiAnceR*). The second adaptation combined the residualized values with called genotypes from rs probes (*EpiAnceR+*).

### Scatterplots

When plotting ancestry PCs against each other, distinct ancestry groups should ideally form dense and separate clusters, while repeated samples from the same individuals should cluster closely together.

**Fig. 1:**
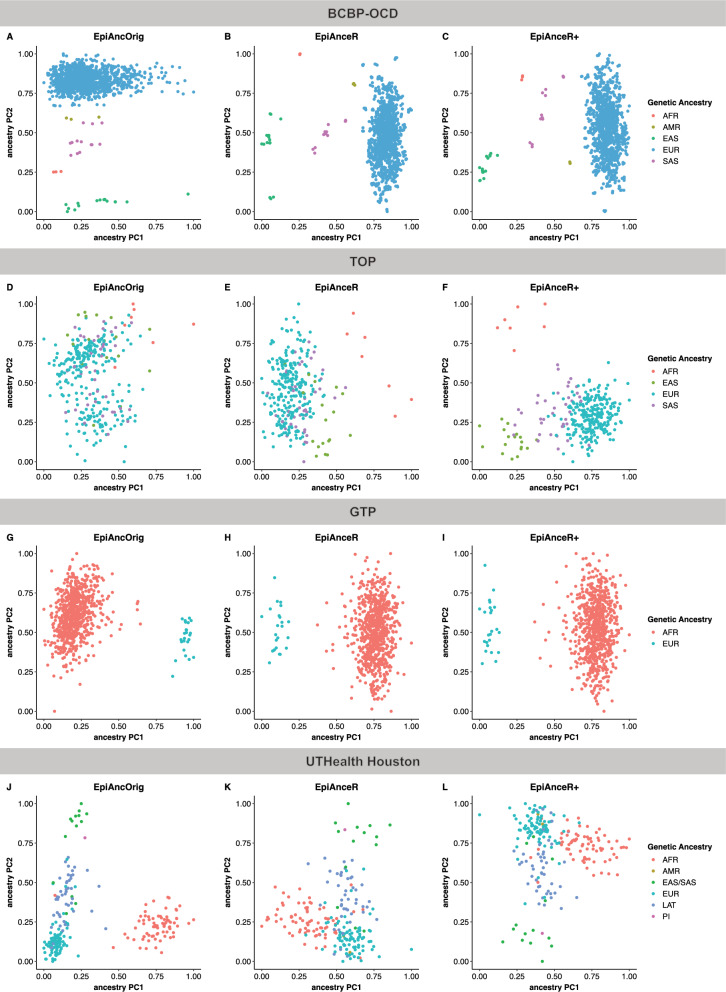
Scatterplots of the first two ancestry PCs plotted against each other, calculated using different approaches. The comparison included the original approach by Barfield et al. (*EpiAncOrig*), the data after residualization for technical and biological factors (*EpiAnceR*), and the data after integrating called genotypes from rs probes with the residualized data (*EpiAnceR+*). *AFR* African, *AMR* American, *EAS* East Asian, *EUR *European, *LAT* Latin American, *PI* Pacific Islander*,* *SAS* South Asian

In the BCBP-OCD cohort (Fig. 1A-C; Figure S1-S3), all approaches visually distinguished ancestry groups in the first two PCs. Notably, while the original approach (*EpiAncOrig*) separated ancestry primarily along the second PC, both adapted approaches achieved separation along the first PC. In the TOP cohort (Fig. 1D-F; Figure S4-S6), the *EpiAncOrig* approach failed to separate ancestry groups. In contrast, *EpiAnceR* successfully differentiated African and East Asian samples, with further improvement using *EpiAnceR+* , separating all ancestry groups. In the GTP cohort, both the *EpiAncOrig* and adapted approaches differentiate ancestry group, with no major observable differences between them (Figures. 1G-I and S7-S9). In the UTHealth Houston cohort, the *EpiAncOrig* approach appears to produce slightly clearer separations and denser clusters compared to the adapted approaches (Figs. 1J-L and S10-S12).

### Association between the ancestry PCs and the ancestry groups

To optimally adjust for ancestry, a strong association between ancestry PCs and ancestry groups is needed, particularly for the first ancestry PC.

**Fig. 2:**
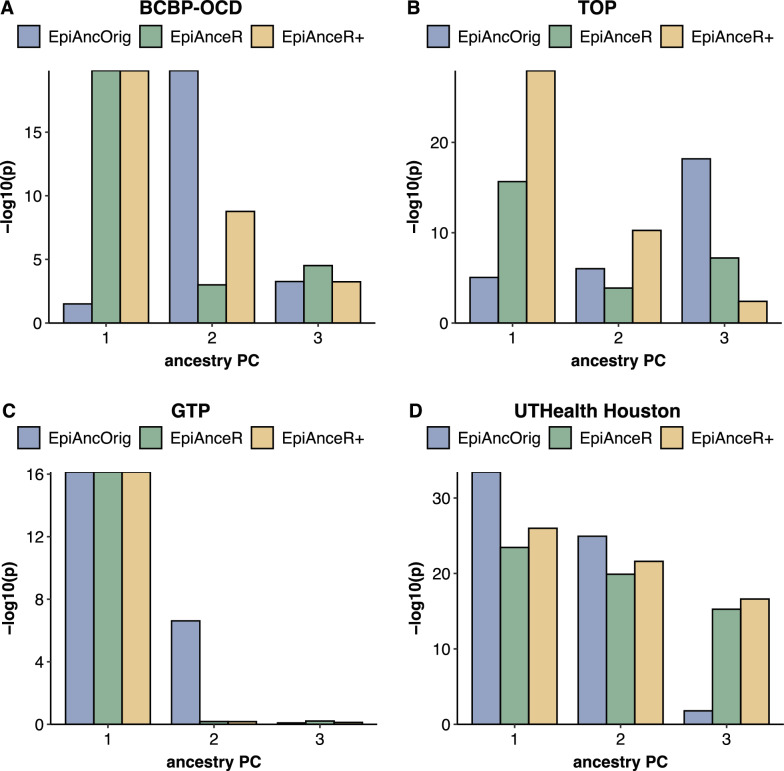
Association between the first three ancestry PCs and the ancestry groups. The comparison included the original approach by Barfield et al. (*EpiAncOrig*), the data after residualization for technical and biological factors (*EpiAnceR*), and the data after integrating called genotypes from rs probes with the residualized data (*EpiAnceR+*)

In the BCBP-OCD cohort (Fig. 2A, Table S1A), both adapted approaches had a much stronger association between the first ancestry PC and the ancestry groups (both p = 1.47E-20) compared to the *EpiAncOrig* approach (p = 0.032), aligning with the observations in the scatterplots (Fig. 1A-C). The *EpiAncOrig* approach showed the strongest association only with the second ancestry PC (p = 1.47E-20), while *EpiAnceR+* (p = 1.68E-09) had a stronger association than *EpiAnceR* (p = 0.001) for the second ancestry PC.

In the TOP cohort (Fig. 2B, Table S1B), the strongest association between the first ancestry PC and the ancestry groups was observed for *EpiAnceR+* (p = 1.15E-22), followed by *EpiAnceR* (p = 2.19E-10), while there was no association with the *EpiAncOrig* approach (p = 1). The *EpiAncOrig* approach showed a significant association only for the third ancestry PC (p = 6.62E-13).

In the GTP cohort, there was no notable difference in the association between the first ancestry PC and ancestry groups (Fig. 2C, Table S1C), while in the UTHealth Houston cohort, the first ancestry PC from the *EpiAncOrig* approach showed stronger associations with ancestry groups than the adapted approaches (Fig. 2D, Table S1D).

### Repeated sample clustering

We next assessed the clustering of repeated samples from the same individuals when plotting ancestry PCs against each other. This analysis was done in both the BCBP-OCD and TOP cohorts that included repeated samples. To quantify clustering, we used silhouette scores (higher scores indicate better clustering) and calculated the 3D distances from the repeated sample to the 3D centroid of the respective individual. Repeated samples should cluster closely together, as genetic ancestry remains consistent over time.

**Fig. 3:**
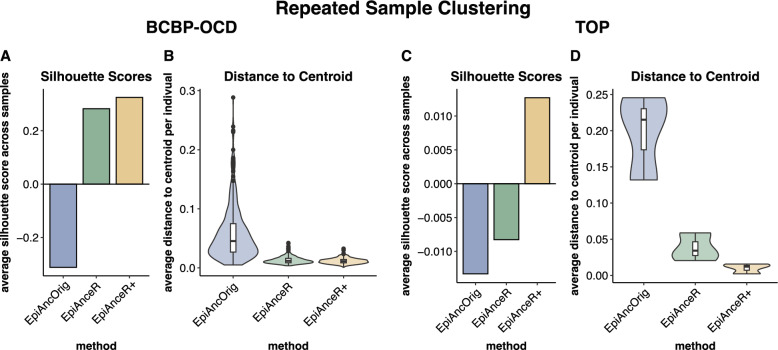
Repeated sample clustering. Clustering performance of repeated samples from individuals was tested using silhouette scores and 3D distance to the individual’s centroid. The comparison included the original approach by Barfield et al. (*EpiAncOrig*), the data after residualization for technical and biological factors (*EpiAnceR*), and the data after integrating called genotypes from rs probes with the residualized data (*EpiAnceR+*)

In the BCBP-OCD cohort, repeated samples clustered more tightly using the adapted approaches compared to the *EpiAncOrig* approach. This was reflected in higher silhouette scores (Fig. 3A, Table S2A) and smaller distances to the individual’s centroid (Fig. 3B, Table S2B). There was little difference between *EpiAnceR* and *EpiAnceR+* .

In the TOP cohort, the *EpiAnceR+* approach resulted in the best clustering, with the highest average silhouette score (Fig. 3C, Table S2A) and the smallest average distance to the centroid (Fig. 3D, Table S2B). However, in the TOP cohort, only three individuals had repeated samples, with each having two samples.

### Ancestry level clustering

Next, we assessed how well different ancestry groups cluster by plotting the first three ancestry PCs against each other. Ideally, samples within the same ancestry group should cluster closely together, while different ancestry groups should be well-separated. Clustering was quantified with silhouette scores and 3D distances both between ancestry clusters and within clusters (from the samples to the respective centroid of the individual).

**Fig. 4:**
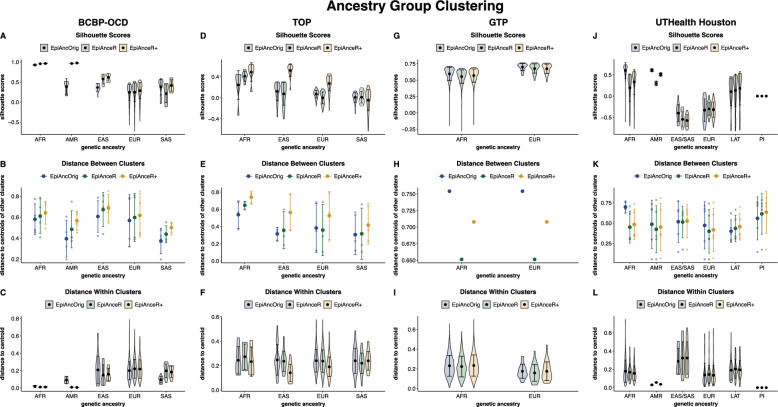
Ancestry group clustering. Clustering performance of samples from different ancestries was tested using silhouette scores and 3D distance to the ancestry group’s centroid. The comparison included the original approach by Barfield et al. (*EpiAncOrig*), the data after residualization for technical and biological factors (*EpiAnceR*), and the data after integrating called genotypes from rs probes with the residualized data (*EpiAnceR+*). *AFR* African, *AMR* American, *EAS* East Asian, EUR: European, *LAT* Latin American, *PI* Pacific Islander, *SAS* South Asian

In the BCBP-OCD cohort, the silhouette scores improved from the *EpiAncOrig* approach (0.2419) to *EpiAnceR* (0.2610) and showed only slight further improvement for *EpiAnceR+* (0.2693) (Fig. 4A, Table S3A-B). The highest mean distances between ancestry clusters were observed with *EpiAnceR+* , followed by *EpiAnceR* and the *EpiAncOrig* approach (Fig. 4B, Table S3C). Distances within clusters presented a mixed picture: while most ancestry groups had tighter clusters with the adapted approaches, the South Asian group showed a wider cluster (Fig. 4C, Table S3C).

In the TOP cohort, the average silhouette scores for *EpiAncOrig* (0.06964) and *EpiAnceR* (0.01417) were lower than for *EpiAnceR+* (0.25019) (Fig. 4D, Table S3A). Clustering varied across ancestry groups, with European, East Asian, and African ancestries showing the best clustering under *EpiAnceR+* , as indicated by higher silhouette scores (Fig. 4D, Table S3B), slightly higher distances between the ancestry clusters (Fig. 4E, Table S3C), and lower distances of samples to the cluster centroid (Fig. 4F, Table S3C) compared to the other approaches.

In the GTP cohort (Fig. 45G-I, Table S3A-C), the *EpiAncOrig* approach produced the highest silhouette score (0.5990), followed by *EpiAnceR+* (0.5732) and *EpiAnceR* (0.5579). This result was primarily driven by larger distances between clusters in the EpiAncOrig approach, followed by *EpiAnceR+* and *EpiAnceR*.

In the UTHealth Houston cohort (Fig. 4J-L, Table S3A-C), the *EpiAncOrig* approach also showed the highest silhouette score (0.07268), compared to *EpiAnceR+* (-0.0567) and *EpiAnceR* (-0.0083). This was mainly due to larger distances between the African ancestry cluster to the other clusters and smaller distances within the Asian cluster under the *EpiAncOrig* approach.

### Comparison with DNA methylation PCs (DNAmPCs) and surrogate variables (SVs)

We compared the performance of *EpiAnceR+* with DNAmPCs and SVs in the BCBP-OCD cohort. *EpiAnceR+* consistently outperformed both approaches. Visually, it produced better clustering of samples (Figure S13), which was reflected in a higher mean Silhouette score (*EpiAnceR+* : 0.293; SVs: -0.226; DNAmPCs: -0.245). Statistically, the first *EpiAnceR+* PC showed a strong association with ancestry groups, whereas only the third DNAmPC and SV demonstrated ancestry association, and with lower significance (Table S1E (supplementary), Figure S14). For repeated samples from the same individual, *EpiAnceR+* again performed better, achieving a higher mean 3D Silhouette score (0.325) compared with DNAmPCs (-0.599) and SVs (-0.603).

### Association of ancestry groups with genotyping PCs

For DNA methylation-based ancestry PCs to effectively capture genetic background information, they should exhibit a strong correlation with genotyping PCs, especially between the first ancestry PC and the first genotyping PC, as both are expected to account for the majority of genetic variation. Pearson correlation analysis was performed between ancestry and genotyping PCs in the **BCBP-OCD** cohort, which had readily available genotyping data.

**Fig. 5:**
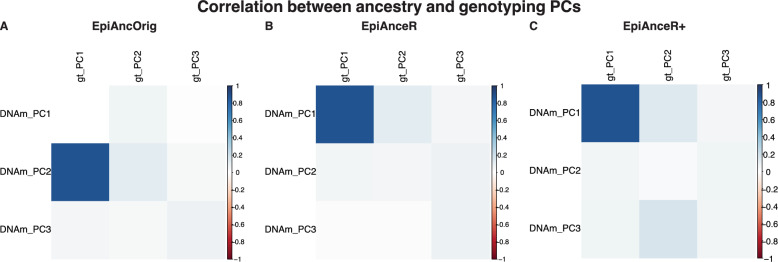
Correlation between the first three ancestry PCs and the first three genotyping PCs. Correlation analyses were performed in the BCBP-OCD cohort. Ancestry PCs are referred to as PCs 1–3 and genotyping PCs as gt_PC1-3. The comparison included the original approach by Barfield et al. (*EpiAncOrig*), the data after residualization for technical and biological factors (*EpiAnceR*), and the data after integrating called genotypes from rs probes with the residualized data (*EpiAnceR+*)

Importantly, both adapted approaches (*EpiAnceR* and *EpiAnceR+*) showed a strong correlation between the first ancestry PC and the first genotyping PC (r = 0.875, Fig. 5B-C). In contrast, the *EpiAncOrig* approach showed no correlation between the first ancestry PC and the first genotyping PC (r = 0.001, Fig. 5A). The mean correlation of the first three genotyping PCs with the first three ancestry PCs was highest for *EpiAnceR+* (mean r = 0.173), followed by *EpiAnceR* (mean r = 0.156), and lowest for the *EpiAncOrig* approach (mean r = 0.146).

In the GTP cohort, partial R^2^ analyses showed that *EpiAnceR+* PC1 captured substantial true ancestry signal (94.4%) but also retained variance explained by residualized covariates (40.5%). PC2 was minimally associated with ancestry (0.5%) or residualized factors (3.4%). PC3 was predominantly explained by residualized variables (49.4%) and showed little ancestry signal (1.1%). These results indicate that, while PC1 largely reflects true ancestry, some PC3 is strongly influenced by residualized technical and biological variation. This pattern is consistent with an unbalanced experimental design that induces correlations between ancestry and residualized factors.

### Ancestry group associations with technical and biological adjustment factors

To assess potential confounding, we tested whether factors adjusted for during residualization (sex, age, cell type proportions, and control probe PCs) were associated with ancestry groups, as such associations could inadvertently reduce true ancestry signals if these factors are unevenly distributed across batches or groups.

In the BCBP-OCD cohort, none of the factors included in the residualization step were associated with ancestry groups (Table S4A). In the TOP cohort, sex, several cell type proportion PCs (1, 2, 4, 5) but only the sixth control probe PC showed significant associations with ancestry groups (Table S4B). In the GTP cohort, age, the second, fourth, and ninth control probe PCs, and the first and second cell type proportion PCs were significantly associated with ancestry groups (Table S4C). In the UTHealth Houston cohort, the first and second cell type PCs were significantly associated with ancestry groups as well, along with the eighth to tenth control probe PCs (Table S4D).

### Practical example

In the UTHealth Houston cohort, we analyzed bipolar case–control status while adjusting for varying numbers of ancestry PCs calculated with *EpiAnceR+* . The genomic inflation factor (λ) is ideally close to 1, as values above 1 may indicate population stratification or other confounding effects.

Without adjusting for any ancestry PCs, λ was 1.087. Adding 2 ancestry PCs reduced λ to 1.023, and adding 5 ancestry PCs further reduced it to 1.008. However, adding 10 ancestry PCs increased λ slightly to 1.056. These results indicate that adjusting for ancestry PCs effectively reduces batch effects introduced by ancestry, with 5 PCs being optimal in this case. Including more PCs than necessary (as with 10 PCs here) can reintroduce noise.

## Discussion

We demonstrated that adjusting for genetic ancestry in DNA methylation studies by residualizing beta values for known technical and biological factors - prior to calculating ancestry principal components from CpGs overlapping with SNPs - and integrating the residualized data with genotype calls from the SNP probes present on the arrays, improves accuracy compared to the method proposed by Barfield et al. (2014) [2].

In the BCBP-OCD and TOP cohorts, we observed the same issues that were reported with the original approach by Barfield et al. (2014) [2]: the first ancestry PC calculated using this approach showed weak associations with the ancestry groups, while stronger associations only emerged for the second or third ancestry PCs. There was a lack of correlation between the first genotyping PC and the first ancestry PC. Thus, these findings suggest that the first ancestry PC calculated with the Barfield et al. [2] approach, and in some cases even the second ancestry PC, mostly capture influences on DNA methylation that are unrelated to ancestry or genetic background.

Several tests were conducted to assess the performance of the adapted approach compared to the original approach. The results indicated that the adapted approach substantially removed technical and biological variance, particularly in the BCBP-OCD and TOP cohorts. Importantly, the association between the first ancestry PC and different ancestry groups was stronger using the adapted approach. While the addition of called genotypes to the residualization step did not substantially affect results in the BCBP-OCD cohort, the TOP cohort showed a stronger association between the first ancestry PC and the ancestry groups when called genotypes were included. This highlights that incorporating called genotypes can improve the robustness, especially in cohorts with larger technical effects.

A key indicator of the adapted approach’s performance is its ability to cluster repeated samples from the same individuals more closely than the original approach. This is demonstrated by higher silhouette scores, a measure of clustering performance, and tighter clustering, indicated by smaller distances to the centroid within each individual’s cluster, in both the BCBP-OCD and TOP cohorts. Since genetic ancestry is consistent over time, repeated samples should cluster tightly together. The tighter clustering observed with the adapted approach aligns with our expectations, as the original approach is affected by technical and biological influences, such as differing plate positions and variations in cell type estimations across samples from the same individual; the adapted approach effectively corrects for these influences, resulting in more accurate ancestry estimations.

In addition to improved individual-level clustering, the different ancestry groups also showed better clustering when plotting ancestry PCs against one another. In the TOP cohort, where batch effects were more pronounced, incorporating called genotypes resulted in higher silhouette scores compared to using the residualized data alone. In contrast, in the BCBP-OCD cohort, where batch effects were less pronounced, the addition of genotypes had minimal impact on clustering performance at the ancestry group level. The tightness of clusters, measured as the distances of samples to their respective ancestry cluster centroids, yielded mixed results across different ancestry groups. This variability may reflect the adapted approach’s ability to capture greater genetic diversity within ancestry groups when confounding factors are appropriately adjusted for.

Finally, the adapted approach demonstrated a strong correlation (r = 0.875) between the first ancestry PCs and the first genotyping PC in the BCBP-OCD cohort, whereas no such correlation was observed with the original approach. The first genotyping PC captures most genetic background, which demonstrates the ability of the first ancestry PC to also capture most of the genetic variability. Moreover, when examining the mean correlation of the first three ancestry PCs with the first three genotyping PCs, the adapted approach including the called genotypes showed the highest correlations of the compared approaches, capturing more true genetic information.

In the BCBP-OCD cohort, *EpiAnceR+* showed clearly superior performance compared with DNA methylation PCs (DNAmPCs) and surrogate variables (SVs), demonstrating stronger statistical associations with ancestry, better clustering of ancestry groups, and improved clustering of repeated samples. Overall, *EpiAnceR+* is more effective at capturing true ancestry effects, whereas DNAmPCs are more likely to capture technical noise and SVs tend to reflect other non-ancestry influences.

The adjusted method did not perform better for the GTP and UTHealth Houston cohorts. Our partial R^2^ analysis in the GTP cohort provides quantitative support for this observation: while *EpiAnceR+* PC1 retained strong ancestry signal (94.4% variance explained by genotype PCs), it also contained substantial variance from residualized covariates (40.5%), and PC3 was largely driven by residualized variables (49.4%) with minimal ancestry signal (1.1%). This pattern is consistent with unbalanced study design, where technical and biological covariates are unevenly distributed across ancestry groups, creating correlations between ancestry and residualized factors. These results indicate that some ancestry effect is removed when correcting for biological and technical factors in the residualizing step, because these factors are themselves associated with ancestry. There is a balance between removing the effects of technical and biological factors and removing ancestry information associated with these factors. If batch effects are not too strong but still associated with ancestry, then ancestry clustering does not improve, as seen in the GTP and UTHealth Houston cohorts. In contrast, substantial improvements are observed in cohorts like the BCBP-OCD cohort, where there is little to no association between ancestry and the factors adjusted for during residualization. Similarly, in the TOP cohort, which has large technical batch effects, residualization had an improving effect despite the removal of some ancestry-related information. The actual performance is sample-specific and depends on the study design, but the method is applicable across a range of sample sizes. Thus, it is important to take more care in designing the studies so that technical factors are not associated with ancestry (by a randomized design, balancing ancestry across plates, slides, and slide positions) and that sex and age are as balanced as possible across ancestries. This makes it possible for the residualization step to effectively remove influencing factors while preserving the ancestry effects.

### Limitations

One limitation is the relatively low overall mean correlation between the first three ancestry PCs and the first three genotyping PCs (mean r = 0.173 for the adapted approach including the genotypic data), suggesting that it does not fully capture all genotyping effects. However, the high correlation between the first ancestry PC and the first genotyping PC (r = 0.875) indicates that the approach effectively captures most of the genetic variation. Despite this, genotyping data remains the gold standard for accurately capturing genetic ancestry and should be used whenever available to adjust for genetic background in DNA methylation studies. Expanding the coverage of Illumina DNA methylation arrays to include more sites overlapping with SNP probes specifically designed to capture ancestry could enhance the ancestry signal derived from these arrays and improve alignment with genotyping PCs; however, in the design of the EPIC v2, a substantial number of SNP-associated methylation probes were removed (selected ancestry-informative probes: 450 K: n = 7295; EPIC v1: n = 3622; EPIC v2: n = 2674).

Another limitation is that the precision of the performance testing may be affected by the fact that our approach was primarily tested on European samples, with fewer available samples from other ancestries. Furthermore, the UTHealth Houston cohort relied on self-reported ancestry data, which is known to be less accurate and may have led to the inclusion of individuals with mixed or misclassified genetic ancestry. Additionally, while "Latino" is officially recognized as an ethnicity rather than a genetic ancestry group, we used it in our analysis as a label for an ancestry cluster. This cluster reflects admixed genetic backgrounds - typically Indigenous American, European, and African - commonly seen in individuals self-identifying as Latino.

Additionally, the longer runtime of our approach presents a practical limitation, primarily due to the detection p-value calculation step. However, this issue can be mitigated by providing pre-calculated detection p-values as input to the function (if already calculated), significantly reducing processing time.

We considered comparing the performance of *EpiAnceR+* with EPISTRUCTURE [4]; however, EPISTRUCTURE has not been updated for the latest arrays and is therefore unlikely to perform reliably on our data.

### Practical implications

*EpiAnceR+* can be used to adjust for ancestry in all samples, even when technical factors correlate with ancestry, as it successfully clustered samples based on ancestry across all tested datasets. Importantly, in cohorts where ancestry is correlated with technical and biological effects, our method ensures that ancestry is not accounted for twice. Unlike the method by Barfield et al. [2], which risks collinearity by adjusting for ancestry alongside confounding factors, our adapted method estimates the remaining ancestry effect while preserving relevant variation.

Compared to EPISTRUCTURE [4], *EpiAnceR+* offers improved usability and compatibility. EPISTRUCTURE is implemented in Python within the GLINT framework and requires multiple preprocessing steps, limiting its integration with standard R-based pipelines. Moreover, it has not been updated since 2017 and does not support the EPIC v2 array. *EpiAnceR+* , by contrast, is R-based and supports all major array types (450 K, EPIC v1, EPIC v2).

Ancestry PCs should be calculated only for samples that have passed general quality control. Based on our experience, including one or two ancestry PCs is often sufficient for most analyses, though the number required depends on cohort diversity. In highly heterogeneous populations, additional ancestry PCs may be necessary. We recommend testing different models (e.g., using 2, 5, or 10 ancestry PCs) and evaluating their performance using QQ plots and genomic inflation factor (λ), as demonstrated in the practical example in the Results section. The optimal number of PCs is the one that brings λ closest to 1.0 and gives QQ plots with no evidence of inflation, indicating adequate correction for population stratification without overfitting.

## Conclusions

Adequate adjustment for genetic ancestry is essential in DNA methylation studies. When genotyping data is unavailable, it has been suggested to use DNA methylation data from Illumina arrays overlapping with SNPs as an alternative. We have improved this approach by accounting for technical and biological influences and incorporating genotypic data from SNP probes, offering increased robustness. Our adapted approach (*EpiAnceR+*) is easy to implement in existing R pipelines, including the ones for the EPIC v2 array, and offers a practical solution for ancestry adjustment.

## Supplementary Information

Overview of Supplementary Materials

Supplementary Figure S1

Supplementary Figure S2

Supplementary Figure S3

Supplementary Figure S4

Supplementary Figure S5

Supplementary Figure S6

Supplementary Figure S7

Supplementary Figure S8

Supplementary Figure S9

Supplementary Figure S10

Supplementary Figure S11

Supplementary Figure S12

Supplementary Figure S13

Supplementary Figure S14

Supplementary Figure S15

Supplementary Table S1

Supplementary Table S2

Supplementary Table S3

## Data Availability

No datasets were generated or analysed during the current study.

## References

[1] Breeze CE, Beck S, Berndt SI, Franceschini N. The missing diversity in human epigenomic studies. Nat Genet. 2022;54(6):737–9. 35681055 10.1038/s41588-022-01081-4 PMC9832920

[2] Barfield RT, Almli LM, Kilaru V, Smith AK, Mercer KB, Duncan R, et al. Accounting for population stratification in DNA methylation studies. Genet Epidemiol. 2014;38(3):231–41. 24478250 10.1002/gepi.21789 PMC4090102

[3] Auton A, Brooks LD, Durbin RM, Garrison EP, Kang HM, et al. A global reference for human genetic variation (Genomes project consortium ). Nature. 2015;526(7571):68–74. 26432245 10.1038/nature15393 PMC4750478

[4] Rahmani E, Shenhav L, Schweiger R, Yousefi P, Huen K, Eskenazi B, et al. Genome-wide methylation data mirror ancestry information. Epigenetics Chromatin. 2017;10:1. 28149326 10.1186/s13072-016-0108-y PMC5267476

[5] Wortinger LA, Stavrum AK, Shadrin AA, Szabo A, Rukke SH, Nerland S, et al. Divergent epigenetic responses to perinatal asphyxia in severe mental disorders. Transl Psychiatry. 2024;8(14):16. 10.1038/s41398-023-02709-7 PMC10774425 38191519

[6] Engh JA, Friis S, Birkenaes AB, Jónsdóttir H, Klungsøyr O, Ringen PA, et al. Delusions are associated with poor cognitive insight in schizophrenia. Schizophr Bull. 2010;36(4):830–5. 19176474 10.1093/schbul/sbn193 PMC2894586

[7] Simonsen C, Sundet K, Vaskinn A, Birkenaes AB, Engh JA, Faerden A, et al. Neurocognitive dysfunction in bipolar and schizophrenia spectrum disorders depends on history of psychosis rather than diagnostic group. Schizophr Bull. 2011;37(1):73–83. 19443616 10.1093/schbul/sbp034 PMC3004191

[8] Mirza S, Lima CNC, Del Favero-Campbell A, Rubinstein A, Topolski N, Cabrera-Mendoza B, et al. Blood epigenome-wide association studies of suicide attempt in adults with bipolar disorder. Transl Psychiatry. 2024;14(1):70. 38296944 10.1038/s41398-024-02760-y PMC10831084

[9] Gillespie CF, Bradley B, Mercer K, Smith AK, Conneely K, Gapen M, et al. Trauma exposure and stress-related disorders in inner city primary care patients. Gen Hosp Psychiatry. 2009;31(6):505–14. 19892208 10.1016/j.genhosppsych.2009.05.003 PMC2785858

[10] Aryee MJ, Jaffe AE, Corrada-Bravo H, Ladd-Acosta C, Feinberg AP, Hansen KD, et al. Minfi: a flexible and comprehensive bioconductor package for the analysis of Infinium DNA methylation microarrays. Bioinformatics. 2014;30(10):1363–9. 24478339 10.1093/bioinformatics/btu049 PMC4016708

[11] Just AC, Heiss JA. ewastools: EWAS Tools. 2023.

[12] Pidsley R, Wong CCY, Volta M, Lunnon K, Mill J, Schalkwyk LC. A data-driven approach to preprocessing Illumina 450K methylation array data. BMC Genomics. 2013;1(14):293. 10.1186/1471-2164-14-293 PMC3769145 23631413

[13] Zheng SC, Webster AP, Dong D, Feber A, Graham DG, Sullivan R, et al. A novel cell-type deconvolution algorithm reveals substantial contamination by immune cells in saliva. Buccal Cervix Epigenomics. 2018;10(7):925–40. 29693419 10.2217/epi-2018-0037

[14] Salas LA, Koestler DC. FlowSorted.Blood.EPIC: Illumina EPIC data on immunomagnetic sorted peripheral adult blood cells. [Internet]. 2024. Available from: https://github.com/immunomethylomics/FlowSorted.Blood.EPIC

[15] Katrinli S, Wani AH, Maihofer AX, Ratanatharathorn A, Daskalakis NP, Montalvo-Ortiz J, et al. Epigenome-wide association studies identify novel DNA methylation sites associated with PTSD: a meta-analysis of 23 military and civilian cohorts. Genome Med. 2024;16(1):147. 39696436 10.1186/s13073-024-01417-1 PMC11658418

[16] Teschendorff AE, Breeze CE, Zheng SC, Beck S. A comparison of reference-based algorithms for correcting cell-type heterogeneity in Epigenome-Wide Association Studies. BMC Bioinform. 2017;18(1):105. 10.1186/s12859-017-1511-5 PMC5307731 28193155

[17] Salas LA, Koestler DC, Butler RA, Hansen HM, Wiencke JK, Kelsey KT, et al. An optimized library for reference-based deconvolution of whole-blood biospecimens assayed using the Illumina HumanMethylationEPIC BeadArray. Genome Biol. 2018;19(1):64. 29843789 10.1186/s13059-018-1448-7 PMC5975716

[18] Houseman EA, Accomando WP, Koestler DC, Christensen BC, Marsit CJ, Nelson HH, et al. DNA methylation arrays as surrogate measures of cell mixture distribution. BMC Bioinform. 2012;13(1):86. 10.1186/1471-2105-13-86 PMC3532182 22568884

[19] Strom NI, Gerring ZF, Galimberti M, Yu D, Halvorsen MW, Abdellaoui A, et al. Genome-wide analyses identify 30 loci associated with obsessive-compulsive disorder [Internet]. medRxiv; 2025 [cited 2025 May 1]. p. 2024.03.13.24304161. Available from: https://www.medrxiv.org/content/. 10.1101/2024.03.13.24304161v2 10.1038/s41588-025-02189-z PMC12165847 40360802

[20] Halvorsen MW, de Schipper E, Bäckman J, Strom NI, Hagen K, Lindblad-Toh K, et al. A burden of rare copy number variants in obsessive-compulsive disorder. Mol Psychiatry. 2025;30(4):1510–7. 39463448 10.1038/s41380-024-02763-7 PMC11919692

[21] Werner MCF, Wirgenes KV, Shadrin A, Lunding SH, Rødevand L, Hjell G, et al. Immune marker levels in severe mental disorders: associations with polygenic risk scores of related mental phenotypes and psoriasis. Transl Psychiatry. 2022;12(1):38. 35082268 10.1038/s41398-022-01811-6 PMC8792001

[22] Nievergelt CM, Maihofer AX, Klengel T, Atkinson EG, Chen CY, Choi KW, et al. International meta-analysis of PTSD genome-wide association studies identifies sex- and ancestry-specific genetic risk loci. Nat Commun. 2019;10(1):4558. 31594949 10.1038/s41467-019-12576-w PMC6783435

[23] Tian Y, Morris TJ, Webster AP, Yang Z, Beck S, Feber A, et al. ChAMP: updated methylation analysis pipeline for Illumina BeadChips. Bioinformatics. 2017;33(24):3982–4. 28961746 10.1093/bioinformatics/btx513 PMC5860089

[24] Hansen KD. IlluminaHumanMethylation450kanno.ilmn12.hg19: Annotation for Illumina’s 450k methylation arrays. 2021.

[25] Pidsley R, Zotenko E, Peters TJ, Lawrence MG, Risbridger GP, Molloy P, et al. Critical evaluation of the Illumina MethylationEPIC BeadChip microarray for whole-genome DNA methylation profiling. Genome Biol. 2016;17(1):208. 27717381 10.1186/s13059-016-1066-1 PMC5055731

[26] Zhang Z, Zeng C, Zhang W. Characterization of the Illumina EPIC array for optimal applications in epigenetic research targeting diverse human populations. Epigenetics Commun. 2022;1(2):7. 10.1186/s43682-022-00015-9 PMC9718568 36466778

[27] Infinium MethylationEPIC v2.0 Product Files [Internet]. [cited 2024 Nov 28]. Available from: https://support.illumina.com/downloads/infinium-methylationepic-v2-0-product-files.html

[28] Rousseeuw PJ. Silhouettes: A graphical aid to the interpretation and validation of cluster analysis. J Comput Appl Math. 1987;1(20):53–65.

[29] Maechler M, Rousseeuw P, Struyf A, Hubert M, Hornik K. Cluster: cluster analysis basics and extensions [Internet]. 2023. Available from: https://CRAN.R-project.org/package=cluster

[30] Wickham H. ggplot2: Elegant Graphics for Data Analysis [Internet]. New York, NY: Springer; 2009 [cited 2023 May 22]. Available from: https://link.springer.com/10.1007/978-0-387-98141-3

[31] Wilke CO. cowplot: Streamlined Plot Theme and Plot Annotations for ‘ggplot2’ [Internet]. 2024. Available from: https://wilkelab.org/cowplot/

[32] Pedersen TL. patchwork: The Composer of Plots [Internet]. 2024. Available from: https://patchwork.data-imaginist.com

[33] Inc PT. Collaborative data science [Internet]. Montreal, QC: Plotly Technologies Inc.; 2015. Available from: https://plot.ly

[34] Vaidyanathan R, Xie Y, Allaire JJ, Cheng J, Sievert C, Russell K. htmlwidgets: HTML Widgets for R [Internet]. 2023. Available from: https://CRAN.R-project.org/package=htmlwidgets

[35] Wei T, Simko V. R package ‘corrplot’: Visualization of a Correlation Matrix [Internet]. 2024. Available from: https://github.com/taiyun/corrplot

