# Supplementary material for: Optimizing genetic ancestry adjustment in DNA methylation studies: a comparative analysis of approaches
Source: Epigenetics Chromatin. 2025 Oct 14;18:69. doi: 10.1186/s13072-025-00627-0 (PMC12522430; doi:10.1186/s13072-025-00627-0)
Supplement: Supplementary file 1 — Overview of Supplementary Materials [file 13072_2025_627_MOESM1_ESM.pdf]

Supplementary Tables

Table S1: Association between ancestry PCs (generated using different approaches) and genetic ancestry groups. A) BCBP-OCD, B) TOP, C) GTP, D) UTHealth Houston, E) Comparison between *EpiAnceR+*, DNA methylation PCs, and surrogate variables. Associations between ancestry PCs and ancestry group were tested using ANOVA when assumptions of normality and variance homogeneity were met. Otherwise, Kruskal-Wallis tests were used.

Table S2: Average (A) silhouette scores and (B) distances from centroid for repeated samples from individuals. To evaluate clustering performance at the individual (repeated samples) level, silhouette scores were calculated using the *cluster* package. In addition, three-dimensional (3D) centroids of repeated sample clusters were calculated. Cluster density was assessed by calculating the mean Euclidean 3D distance from each sample to the centroid of its respective cluster.

Table S3: Average silhouette scores (A) across ancestries, B) within ancestries) and (C) distances from centroid for samples from ancestry group. To evaluate clustering performance at the group level, silhouette scores were calculated using the *cluster* package. In addition, three-dimensional (3D) centroids of ancestry group clusters were calculated. Cluster density was assessed by calculating the mean

Euclidean 3D distance from each sample to the centroid of its respective group. To determine the separation between ancestry group clusters, a distance matrix was generated to measure the distances between the centroids of different ancestry groups.

Table S4: Association of genetic ancestry groups with technical and biological factors used to residualised ancestry data. A) BCBP-OCD, B) TOP, C) GTP, D) UTHealth Houston. Associations between ancestry groups and residualization factors were tested using linear models (two groups) or ANOVA (multiple groups). For control probe and cell type PCs in datasets with repeated samples, linear mixed-effect models with a random intercept were used. Model fit was assessed via likelihood ratio tests comparing models with and without ancestry group as a predictor.

Supplementary Figures

All Supplementary Figures in html format are best viewed using the Firefox browser.

Figure S1: 3D Plot of the first three ancestry principal components using the EpiAncOrig approach in the BCBP-OCD cohort. AFR: African, AMR: American, EAS: East Asian, EUR: European, SAS: South Asian

Figure S2: 3D Plot of the first three ancestry principal components using the EpiAnceR approach in the BCBP-OCD cohort. AFR: African, AMR: American, EAS: East Asian, EUR: European, SAS: South Asian

Figure S3: 3D Plot of the first three ancestry principal components using the EpiAnceR+ approach in the BCBP-OCD cohort. AFR: African, AMR: American, EAS: East Asian, EUR: European, SAS: South Asian

Figure S4: 3D Plot of the first three ancestry principal components using the EpiAncOrig approach in the TOP cohort. AFR: African, EAS: East Asian, EUR: European, SAS: South Asian

Figure S5: 3D Plot of the first three ancestry principal components using the EpiAnceR approach in the TOP cohort. AFR: African, EAS: East Asian, EUR: European, SAS: South Asian

Figure S6: 3D Plot of the first three ancestry principal components using the EpiAnceR+ approach in the TOP cohort. AFR: African, EAS: East Asian, EUR: European, SAS: South Asian

Figure S7: 3D Plot of the first three ancestry principal components using the EpiAncOrig approach in the GTP cohort

Figure S8: 3D Plot of the first three ancestry principal components using the EpiAnceR approach in the GTP cohort

Figure S9: 3D Plot of the first three ancestry principal components using the EpiAnceR+ approach in the GTP cohort

Figure S10: 3D Plot of the first three ancestry principal components using the EpiAncOrig approach in the UTHealth Houston cohort. AFR: African, AMR: American, EAS: East Asian, EUR: European, LAT: Latin American, PI: Pacific Islander, SAS: South Asian

Figure S11: 3D Plot of the first three ancestry principal components using the EpiAnceR approach in the UTHealth Houston cohort. AFR: African, AMR: American, EAS: East Asian, EUR: European, LAT: Latin American, PI: Pacific Islander, SAS: South Asian

Figure S12: 3D Plot of the first three ancestry principal components using the EpiAnceR+ approach in the UTHealth Houston cohort. AFR: African, AMR: American, EAS: East Asian, EUR: European, LAT: Latin American, PI: Pacific Islander, SAS: South Asian

Figure S13: Scatterplots of the first two ancestry PCs plotted against each other, calculated using different approaches in the BCBP-OCD cohort. The comparison included DNA methylation Principal Components (DNAmPCs), Surrogate Variables (SVs), and *EpiAnceR+*. AFR: African, AMR: American, EAS: East Asian, EUR: European, SAS: South Asian.

Figure S14: Association between the first three PCs/SVs and the ancestry groups in the BCBP-OCD cohort. The comparison included DNA methylation Principal Components (DNAmPCs), Surrogate Variables (SVs), and *EpiAnceR+*.
