# Supplementary figures and images for: Optimizing genetic ancestry adjustment in DNA methylation studies: a comparative analysis of approaches
Source: Epigenetics Chromatin. 2025 Oct 14;18:69. doi: 10.1186/s13072-025-00627-0 (PMC12522430; doi:10.1186/s13072-025-00627-0)

# BCBP-OCD

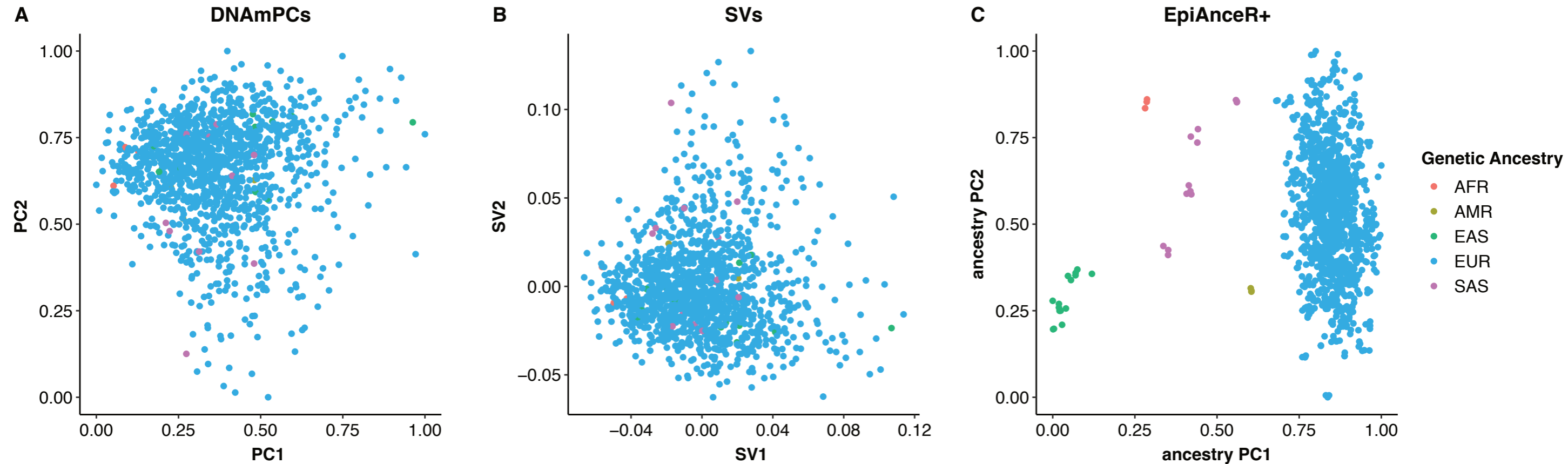

Supplement: Supplementary file 14 — Supplementary Figure S13 [file 13072_2025_627_MOESM14_ESM.pdf]

# Ancestry PCs ~ Ancestry Categories

SVs DNAmPCs EpiAnceR+

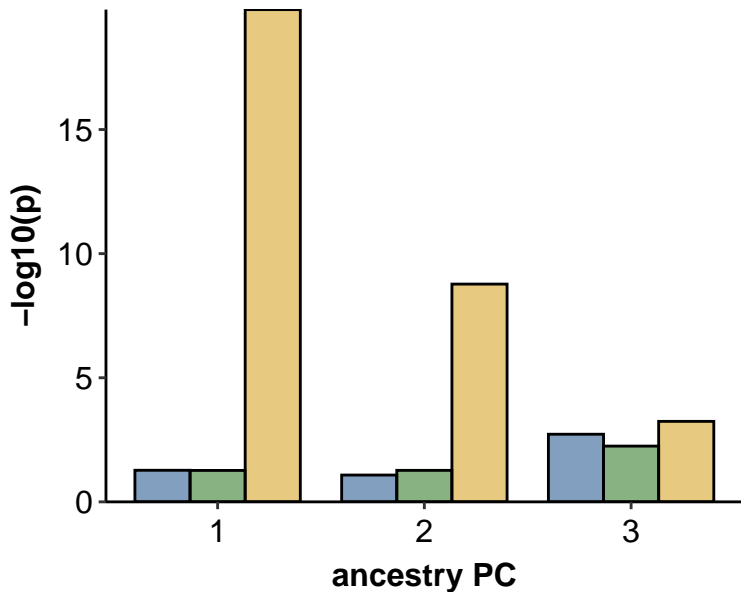

Supplement: Supplementary file 15 — Supplementary Figure S14 [file 13072_2025_627_MOESM15_ESM.pdf]
